# Modification and Validation of the System Causability Scale Using AI-Based Therapeutic Recommendations for Urological Cancer Patients: A Basis for the Development of a Prospective Comparative Study

**DOI:** 10.3390/curroncol31110520

**Published:** 2024-11-11

**Authors:** Emily Rinderknecht, Dominik von Winning, Anton Kravchuk, Christof Schäfer, Marco J. Schnabel, Stephan Siepmann, Roman Mayr, Jochen Grassinger, Christopher Goßler, Fabian Pohl, Peter J. Siska, Florian Zeman, Johannes Breyer, Anna Schmelzer, Christian Gilfrich, Sabine D. Brookman-May, Maximilian Burger, Maximilian Haas, Matthias May

**Affiliations:** 1 Department of Urology, Caritas St. Josef Hospital, University of Regensburg,93053 Regensburg, Germany; erinderknecht@csj.de (E.R.); mschnabel@csj.de (M.J.S.); rmayr@csj.de (R.M.); cgossler@csj.de (C.G.); jbreyer@csj.de (J.B.); mburger@caritasstjosef.de (M.B.); mhaas@csj.de (M.H.); 2 Department of Urology, St. Elisabeth Hospital Straubing, 94315 Straubing, Germany; dominik.vonwinning@klinikum-straubing.de (D.v.W.); anton.kravchuk@klinikum-straubing.de (A.K.); stephan.siepmann@klinikum-straubing.de (S.S.); anna.schmelzer01@stud.uni-goettingen.de (A.S.); christian.gilfrich@klinikum-straubing.de (C.G.); 3 Department of Radiotherapy, Straubing Hospital Medical Care Centre, 94315 Straubing, Germany; christof.schaefermbm@mvz-klinikum-straubing.de; 4 Department of Hematology and Oncology, Straubing Hospital Medical Care Centre, 94315 Straubing, Germany; jochen.grassinger@mvz-klinikum-straubing.de; 5 Department of Radiotherapy, University Hospital Regensburg, 93053 Regensburg, Germany; fabian.pohl@ukr.de; 6 Department of Internal Medicine III, University Hospital Regensburg, 93053 Regensburg, Germany; peter.siska@klinik.uni-regensburg.de; 7 Center for Clinical Studies, University Hospital Regensburg, 93053 Regensburg, Germany; florian.zeman@klinik.uni-regensburg.de; 8 Department of Urology, Ludwig-Maximilians-University (LMU), 80539 Munich, Germany; sabine.brookman-may@email.de

**Keywords:** artificial intelligence integration, large language models, multidisciplinary tumor boards, non-inferiority CONCORDIA Trial, SCS, urological cancer treatment, validation study, clinical decision support, artificial neural network

## Abstract

The integration of artificial intelligence, particularly Large Language Models (LLMs), has the potential to significantly enhance therapeutic decision-making in clinical oncology. Initial studies across various disciplines have demonstrated that LLM-based treatment recommendations can rival those of multidisciplinary tumor boards (MTBs); however, such data are currently lacking for urological cancers. This preparatory study establishes a robust methodological foundation for the forthcoming CONCORDIA trial, including the validation of the System Causability Scale (SCS) and its modified version (mSCS), as well as the selection of LLMs for urological cancer treatment recommendations based on recommendations from ChatGPT-4 and an MTB for 40 urological cancer scenarios. Both scales demonstrated strong validity, reliability (all aggregated Cohen’s K > 0.74), and internal consistency (all Cronbach’s Alpha > 0.9), with the mSCS showing superior reliability, internal consistency, and clinical applicability (*p* < 0.01). Two Delphi processes were used to define the LLMs to be tested in the CONCORDIA study (ChatGPT-4 and Claude 3.5 Sonnet) and to establish the acceptable non-inferiority margin for LLM recommendations compared to MTB recommendations. The forthcoming ethics-approved and registered CONCORDIA non-inferiority trial will require 110 urological cancer scenarios, with an mSCS difference threshold of 0.15, a Bonferroni corrected alpha of 0.025, and a beta of 0.1. Blinded mSCS assessments of MTB recommendations will then be compared to those of the LLMs. In summary, this work establishes the necessary prerequisites prior to initiating the CONCORDIA study and validates a modified score with high applicability and reliability for this and future trials.

## 1. Introduction

The increasing integration of artificial intelligence (AI) into healthcare has recently gained considerable attention, sparking widespread discussions, research, and early adoption. This momentum is driven by its significant potential across multiple domains within the medical sector [1,2,3]. Among the most groundbreaking developments are Generative Pre-trained Transformers (GPTs) and, more broadly, Large Language Models (LLMs), a critical branch of AI and machine learning (ML). These models, powered by advanced ML algorithms, can generate and interpret text without the need for prior linguistic preprocessing, such as traditional Natural Language Processing. Consequently, they hold vast potential for applications in both clinical practice and academic research within the medical field [4,5].

While GPT-2 was introduced in 2018, it was the launch of OpenAI’s ChatGPT on 30 November 2022 that catapulted LLMs into mainstream consciousness, driving global interest and adoption [6]. In healthcare, the impact of AI-generated decisions, recommendations, or outcomes is profound, depending on the specific context and application. However, for these models to be safely and effectively integrated into routine clinical practice, several challenges must be addressed. These include ensuring accuracy, transparency, and accountability, alongside managing ethical concerns and maintaining the central role of the human physician, who bears ultimate responsibility for clinical decisions. As a result, the implementation of these models in medicine remains in its early stages, with significant validation still required for widespread adoption.

A recent international survey of 456 urologists revealed that 53% perceived limitations in LLM usability within clinical and academic settings. The most cited concerns included inaccurate responses (45%), lack of specificity (42%), and inconsistent answers (26%) [7]. Nonetheless, 56% of respondents believed that ChatGPT and other LLMs hold potential value for clinical decision-making, with approximately 20% having already incorporated ChatGPT into their workflows [7]. With that said, 30% of urologists indicated potential applications for LLMs in selecting appropriate treatment options [7]. Multidisciplinary tumor boards (MTBs) serve as a critical component in delivering high-quality, guideline-based care for urological cancer patients through consensus-driven therapy recommendations [8,9]. However, these often-weekly meetings pose substantial time management challenges for clinicians, who are already intended to meet both clinical and academic needs [8,9,10]. Although a few studies have examined the integration of LLMs into MTBs for non-urological cancers, these investigations also highlight current limitations regarding the safe and effective use of such models [11,12,13,14,15,16,17,18,19,20].

However, to the best of our knowledge, no studies have been conducted in the context of genitourinary cancers (GUCs) or compared the blinded recommendations of LLMs with those of an actual MTB [11,13,14,21].

The present study serves as a preparatory investigation to lay the groundwork for a prospective trial aimed at determining whether LLM-generated treatment recommendations for genitourinary cancers (GUCs) can match those of an interdisciplinary MTB comprising specialists from urology, oncology, radiotherapy, and nuclear medicine/radiology.

To assess the quality of AI-generated explanations, especially in the context of scientific model development, Holzinger et al. introduced the System Causability Scale (SCS) in 2020 [22]. The SCS quantifies explainability based on responses to 10 questions, each rated on a 5-point Likert scale. Its simplicity and status as a standardized tool make it highly useful for evaluating AI- and LLM-generated explanations [22].

This study’s objectives include (1) the adaptation of the SCS for specific oncological contexts, (2) the validation of this adapted scale, (3) the Delphi-based selection of LLMs, (4) the determination of a non-inferiority threshold for recommendations, and (5) the sample size calculation for the prospective CONCORDIA study.

## 2. Materials and Methods

### 2.1. Planned Prospective Trial

The proposed study is a prospective investigation designed to examine whether real MTBs can be equivalently replaced by LLMs. The study, titled “Concordance Study on Urological Tumor Boards and Large-Language-Model Substitutes” (CONCORDIA Study), will seek ethical approval and formal registration with the German Clinical Trials Register (Deutsches Register Klinischer Studien, DRKS). It will be conducted at two German hospitals: St. Josef Medical Center (University of Regensburg) and St. Elisabeth Hospital Straubing. These institutions will provide case scenarios of GUC patients, reflecting the typical distribution of tumor entities handled by their respective MTBs.

The primary aim of the study is to compare the blinded therapeutic recommendations of a real MTB, comprising specialists in urology, oncology, radiation therapy, and nuclear medicine, with the recommendations from two selected, publicly available LLMs. This study follows a non-inferiority design, evaluating the performance of LLMs against the real MTB. Study outcomes will be assessed using a modified score (mSCS).

The following key objectives of this preparatory study will be addressed methodologically in subsequent sections: (1) Selection of two appropriate LLMs for comparison with the MTB; (2) Development of standardized prompts for data input on GUC patients and the creation of a uniform recommendation matrix to ensure blinded assessment; (3) Modification and validation of the newly developed mSCS using a cohort of 40 urological tumor patients across various organ-specific cancers; (4) Biometric sample size planning for the prospective trial, preceded by a moderated Delphi process to determine the acceptable non-inferiority threshold for LLM performance compared to the MTB; and (5) Precise documentation of statistical methods to validate the mSCS and compare results between the groups (MTB vs. LLM).

To streamline this preparatory study, the therapeutic recommendations from the real MTB were compared only with those from the premium version of the top-performing LLM. The second LLM will be tested in the main trial using the non-inferiority threshold derived from this preparatory comparison. Ethical approval for this preparatory study was obtained (UKR-EK-24-3835-104). The methodological details are presented in the following sections.

### 2.2. Selection of Two Appropriate LLMs for Comparison with the MTB (1)

The selection of LLMs was made through a consensus among the study authors (ER, MH, DvW, AK, CGo, and MM) using a four-stage Delphi method moderated by MS. The process included the following: Round 1: Identification of all available LLMs and discussion of their theoretical suitability for the study; Round 2: Review of preliminary results based on unspecific German-language prompts applied to virtual GUC case scenarios for each LLM; Round 3: Secret voting among the six panelists, with two points awarded for the most suitable LLM and one point for the second choice (a maximum of twelve points per LLM, with eighteen points total from all panelists); Round 4: A final moderated discussion of the results, leading to consensus on the two LLMs selected for the study.

The selection criteria included (1) availability, (2) suitability for answering medical queries, and (3) response quality. LLMs considered in this process included ChatGPT-4, ChatGPT-3.5 (both OpenAI), Claude 3.5 Sonnet (Anthropic), Copilot (Microsoft), Gemini (Google), Llama 3 (Meta), and Med-PaLM2 (Google). Panelists were also encouraged to favor LLMs utilizing different transformer-based architectures designed for natural language processing tasks.

### 2.3. Development of Standardized Prompts for Data Input on GUC Patients and the Creation of a Uniform Recommendation Matrix for Both LLMs and the MTB to Facilitate Blinded Assessment (2)

German-language prompts were chosen for querying the LLMs to avoid translation errors and ensure direct comparability with the German-language recommendations of the real MTB. The initial prompts were developed by MM based on previous LLM queries and relevant studies [11,12,13,14,15,16,18,19]. These prompts were then tested in a multi-stage process by the working group (ER, MH, DvW, and AK) using the two selected LLMs. Based on the results, the prompts were refined and optimized for consistency and accuracy.

The working group also discussed formal and linguistic adjustments needed to ensure that the recommendations from both the MTB and LLMs could be sufficiently blinded for evaluation. The recommendation matrix was developed through iterative testing, involving pilot studies and refinements to ensure that evaluators remained blinded to the source of recommendations. Each step aimed to maintain an objective comparison between LLM and MTB outputs. This resulted in the development of a clear recommendation matrix. The criteria for finalizing the prompts included (a) the clinical relevance of the recommendations, (b) the consideration of key patient characteristics across the five main GUC types (prostate cancer, bladder cancer, kidney cancer, testicular cancer, and penile cancer), (c) the inclusion of a multidisciplinary treatment perspective, (d) the reference to current evidence, and (e) the ability to offer alternative therapeutic strategies.

### 2.4. Modification and Validation of the Newly Developed mSCS Using a Cohort of 40 Patients with Varying Organ-Specific GUCs (3)

The SCS is a metric for evaluating LLM recommendations. It is calculated by assigning a score between 1 and 5 on a Likert scale to each of the 10 items it comprises. These items are listed in the second column of Table 2. The possible ratings are 1 = strongly disagree, 2 = disagree, 3 = neutral, 4 = agree, and 5 = strongly agree. The ratings for the 10 categories are summed, and the resulting score is obtained by dividing the sum by 50. This yields scores ranging from 0.2 to 1.0, with 1.0 representing the optimal result [22].

The 10 items of the original SCS [22] were reviewed by two uro-oncology experts (MM and CGo) and assessed for their applicability to the study. The experts proposed modifications of items to improve the tool for evaluating treatment recommendations. The items were revised for the mSCS, but the number of items and the consecutive calculation of the score correspond exactly to the original SCS.

Forty case scenarios, encompassing the five main GUCs (16 prostate cancer, 9 bladder cancer, 7 kidney cancer, 4 testicular cancer, and 4 penile cancer cases), were presented to the real MTBs in Regensburg and Straubing (20 per site) and also submitted to the top-rated LLM, based on the Delphi process. Recommendations from both the MTB and the LLM were rated using the SCS by two independent uro-oncologists (ER and MH for Regensburg; DvW and AK for Straubing). The same raters applied the mSCS 14 days later with the same recommendations, with the sequence of case scenarios altered. Any discrepancies between the two raters were resolved by a third adjudicator (CGo in Regensburg; MM in Straubing).

Following the two rating rounds, all four raters (ER, MH, DvW, and AK) were asked to assess the clinical applicability of the mSCS compared to the original SCS using a 5-point Likert scale (1 = severe deterioration, 2 = deterioration, 3 = equality, 4 = improvement, and 5 = strong improvement). The four ratings were combined into one rating for further analysis by determining the modal value. For the statistical analysis, the comparative SCS item was always assigned the value 3.

To comply with data protection guidelines, the case scenarios were realistic but fictitious. These scenarios were developed based on the real patient cases typically presented at the MTBs and were created by experienced uro-oncologists (CGo in Regensburg, MM in Straubing). Each case was formatted as a table in bullet-point form, mirroring the style used in real-life MTB presentations.

### 2.5. Biometric Sample Size Planning for the Prospective Trial, Preceded by a Moderated Delphi Process with the Entire Study Team to Establish What Level of Difference in the mSCS, Derived from Preliminary Study Results, Would Still Be Considered Non-Inferior for LLMs Compared to the MTB (4)

Biometric sample size planning was conducted by a statistician experienced in prospective study design (FZ). The mean mSCS results for the LLM and MTB, along with their respective standard deviations, were used to estimate the expected variance. A *t*-test was used to compare the two groups, with power set at 90% (beta = 0.1) and a one-sided 2.5% level of significance (alpha = 0.025), adjusted by Bonferroni correction since two LLMs were compared against the real MTB (adjusted *p*-value: 0.0125).

The non-inferiority margin for the LLMs, indicating acceptable performance compared to the MTB, was determined by the study authors (ER, MH, DvW, AK, CGo, and MM) through a second four-round Delphi process moderated by author MS. The process included the following: Round 1: Presentation of four differences in mSCS, proposed by the moderator, that were still associated with non-inferiority (0.05, 0.1, 0.15, and 0.2); Round 2: A group discussion on the clinical implications of these four thresholds, based on five GUC scenarios prepared by the moderator; Round 3: Secret voting among the six panelists, with two points awarded for the most suitable difference and one point for the second choice (a maximum of twelve points per cutoff, with eighteen points total from all panelists); Round 4: A final moderated discussion of the results, leading to a consensus on the difference at which clinical non-inferiority of the LLMs compared to the real MTB can still be acknowledged.

### 2.6. Precise Documentation and Listing of Statistical Methods to Validate the mSCS and Compare Results Between the Groups (MTB vs. LLM) (5)

Interrater reliability was assessed using Cohen’s kappa coefficient [23,24]. The intraclass correlation coefficient (ICC) was calculated as an additional reliability measure, confirming substantial agreement among raters and complementing the results obtained from Cohen’s kappa [25]. ICC calculations were based on a mean rating (k = 2), absolute agreement, and a 2-way random-effects model. To simplify analysis for kappa calculation, the 5-point Likert scale ratings for the 10 SCS and mSCS items were dichotomized. Scores differing by more than 1 point were labeled as ‘disagree’, while scores within ±1 were labeled ‘agree’. Reliability was tested for each item in both the SCS and mSCS for the MTB and LLM, and pooled analyses were conducted. The interpretation of Cohen’s kappa (K) can be classified based on the guidelines established by Landis and Koch, who propose the following framework for interpreting the strength of agreement: <0.00: poor agreement, 0.00–0.20: slight agreement, 0.21–0.40: fair agreement, 0.41–0.60: moderate agreement, 0.61–0.80: substantial agreement, and 0.81–1.00: almost perfect agreement [26]. The ICC was interpreted using the following classification of reliability proposed by Koo and Li [27]: <0.5: poor, 0.5–0.75 moderate, 0.75–0.9 good, and >0.9 excellent.

The validity of the mSCS was assessed by comparing its internal consistency with that of the original SCS using Cronbach’s Alpha [28]. While there are different interpretations of Cronbach’s Alpha (α) in the literature [29], we adhere to the commonly used structure as follows: <0.5: unacceptable, 0.50–0.59: poor, 0.60–0.69: questionable, 0.70–0.79: acceptable, 0.80–0.89: good, and ≥0.9: excellent.

Consensus judgments were used in both systems for this analysis. Differences in clinical applicability between the SCS and mSCS were tested for significance using the Wilcoxon signed-rank test.

All *p*-values were two-tailed, and statistical significance was set at *p* ≤ 0.05. Statistical analyses were performed using SPSS 29.0 (IBM Corp., Armonk, NY, USA).

## 3. Results

### 3.1. Selection of Two Appropriate LLMs for Comparison with the MTB (1)

As part of the Delphi process, ChatGPT-4 and Claude 3.5 Sonnet were selected as the most suitable LLMs.

The distribution of points in the anonymous voting between the LLMs using the Delphi method (round 3) showed the following results: ChatGPT-4 received 11 points, Claude 3.5 Sonnet received 5 points, and ChatGPT-3.5 received 2 points. All other LLMs under consideration received no points.

The moderated discussion revealed the following reasons for the low scores achieved by the other LLMs: The response quality from Copilot, as well as ChatGPT-3.5, appeared inferior in test inputs. Gemini did not sufficiently adhere to the required formal conditions of the recommendations in test inputs. Llama 3 was excluded due to its lack of availability in Europe and the frequent issuance of the error message “Sorry, I can’t help you” in reference to a doctor’s consultation. Med-PaLM2 was not sufficiently available.

### 3.2. Development of Standardized Prompts for Data Input on Urological Tumor Patients and the Creation of a Uniform Recommendation Matrix for Both LLMs and the MTB to Facilitate Blinded Assessment (2)

The developed prompt is shown in Table 1. The individual components of the prompt have been assigned to corresponding objectives in the table. They are color-coded according to the following scheme: task (yellow), information provided (green), request for completeness and indication of preferred option (gray), geographical categorization (blue), and formal requirements (purple).

The following matrix was created to enable a blinded rating of the recommendations:

(1) Preferred therapy recommendation (if available);
(2) Therapy alternatives;
(3) Justification of the recommendations;
(4) Supportive measures/supplementary therapies;
(5) Further information/explanations.

The content of the corresponding MTB or LLM recommendation was manually inserted into the matrix in bullet points. The bullet point approach ensures that possible recurring phrases or ways of formulating do not invalidate the blinding. The recommendations focused exclusively on tumor therapy, while guidance on other coexisting conditions unrelated to the tumor was excluded.

### 3.3. Modification and Validation of the Newly Developed mSCS Using a Cohort of 40 GUC Patients with Varying Organ-Specific Cancers (3)

Table 2 shows the SCS and the mSCS. All items, except item 4, were modified.

**Table 2:** Items of original System Causability Scale (SCS) and modified SCS (mSCS).

Item	SCS	mSCS
1	I found that the recommendation included all relevant known causal factors with sufficient precision and granularity.	I found that the recommendation included all relevant patient-specific factors (individual patient data such as individual tumor stages, previous treatments, and specific health conditions) with sufficient precision and granularity.
2	I understood the explanations within the context of my work.	I found the quality and representativeness of the recommendations, particularly in relation to oncological scenarios, sufficient.
3	I could change the level of detail on demand.	I found that all reasonable treatment alternatives were specified.
4	I did not need support to understand the explanations.	I did not need support to understand the explanations.
5	I found the explanations helped me to understand causality	I found that the recommendation was explained and made transparent.
6	I was able to use the explanations with my knowledge base.	I found the recommendation to be consistent with current clinical guidelines.
7	I did not find inconsistencies between explanations.	I did not find inconsistencies between explanations/recommendations.
8	I think that most people would learn to understand the explanations very quickly.	I think that most healthcare professionals would learn to understand the explanations very quickly.
9	I did not need more references in the explanations, e.g., medical guidelines and regulations.	I found the recommendation demonstrates access to the latest research and clinical guidelines.
10	I received the explanations in a timely and efficient manner	I found the quality of interaction (ease of use and accessibility) sufficient.

### 3.4. Biometric Sample Size Planning for the Prospective Trial, Preceded by a Moderated Delphi Process with the Entire Study Team to Establish What Level of Difference in the mSCS, Derived from Preliminary Study Results, Would Still Be Considered Non-Inferior for LLMs Compared to the MTB (4)

After evaluating the LLM and MTB recommendations for the 40 sample tumor cases using the mSCS, the recommendations were compared with the consecutive ratings. Detailed discussions were held to determine which differences in the mSCS corresponded to which differences in the content of recommendations, especially regarding clinical implications. Based on these discussions, barriers that could generally be considered meaningful non-inferiority measures were assessed. This resulted in the considered non-inferiority cutoffs at differences of 3 points, 5 points, 8 points, or 10 points, based on a maximum score of 50 (corresponding to a threshold of 0.05, 0.1, 0.15, and 0.2).

In the following anonymous voting as part of the Delphi process, the non-inferiority threshold of 0.15 difference in mSCS received the highest score of nine points. The thresholds 0.1, 0.05, and 0.2 received five, three, and one point respectively. Finally, another moderated discussion was held regarding the best-scoring non-inferiority threshold of 0.15, in which it was jointly agreed that this maximum difference in mSCS clinically represents a non-inferiority of the recommendation quality. To put this abstract number into a concrete context, the threshold of 0.15 corresponds to an absolute difference of eight points on the SCS and mSCS scales, respectively. For example, if a rater strongly disagreed with the LLM’s recommendation on two items but strongly agreed with the real MTB’s recommendation on those same items, the LLM’s recommendation would be considered inferior. Naturally, the total of eight points may also result from differences across other items.

The mean value of the mSCS scores obtained was 0.992 ± 0.013 for the MTB recommendations. There was a slight inferiority in the mean mSCS of the recommendations of the LLM, which was 0.897 ± 0.144. To show the non-inferiority of the LLM compared to the MTB with an expected difference of Δ = 0.095 ± 0.1445 between both assessments (paired design) at a non-inferiority margin of 0.15 with a power of 90% (beta = 0.1) at a one-sided 1.25% level of significance, a total of 87 cases are needed for statistical analyses (overall *p*-value: 0.025 (one-sided)).

To account for potential dropouts or missing data, we increased the sample size by 25%, targeting 109 participants. One additional case was included to achieve an even number for the bicentric study, ensuring equal distribution across centers. This resulted in a final sample size of 110 cases for the planned prospective study.

### 3.5. Validation of the mSCS and Comparison Between the Groups (MTB vs. LLM) (5)

#### 3.5.1. Interrater Reliability

To assess the agreement between the two independent raters, Cohen’s Kappa and ICC were calculated. The kappa (Κ) values are shown in Table 3a. The ICC values are shown in Table 3b.

Regarding the SCS rating of the MTB recommendations, kappa values of 0.7 to 1.0 (*p* < 0.001) were obtained for the individual items. The pooled analysis resulted in Κ = 0.90 (*p* < 0.001). The corresponding ICC values were 0.83 to 1.0 (*p* < 0.001) for the individual items and 0.95 for the pooled analysis. With regard to the SCS rating of the LLM recommendations, kappa values of 0.65 to 0.90 (*p* < 0.001) were obtained for the individual items. The pooled analysis resulted in Κ = 0.74 (*p* < 0.001). The corresponding ICC values were 0.83 to 0.95 (*p* < 0.001) for the individual items and 0.85 for the pooled analysis. In summary, substantial to almost perfect interrater reliability was shown using Cohen’s Kappa for the SCS across all items. Good to excellent interrater reliability was shown using ICC for the SCS across all items.

For the mSCS ratings regarding the MTB recommendation, for all items, the Kappa values were at least Κ = 0.75, indicating at least substantial agreement. In line with this, the ICC values were at least 0.86, which corresponds to good interrater reliability. For the mSCS ratings regarding the LLM recommendation, slightly more dispersion was observed. The lowest kappa value obtained was Κ = 0.65, which also indicates a substantial agreement. The lowest ICC value obtained was 0.79, which also indicates good reliability. In the pooled analysis of interrater reliability across all items of the mSCS, Κ = 0.95 (*p* < 0.001) and ICC 0.97 (*p* < 0.001) were obtained for the MTB recommendations, and Κ = 0.81 (*p* < 0.001) and ICC 0.89 (*p* < 0.001) for the LLM recommendations (Table 3).

#### 3.5.2. Agreement Between SCS and mSCS

Agreement of the consensus ratings between SCS and mSCS in dichotomized form was calculated using Cohen’s kappa (K). The ratings of the MTB recommendations exhibited an almost perfect agreement of K = 0.96 (*p* < 0.001). With regard to the ratings of the LLM recommendations, there was an almost perfect agreement of K = 0.88 (*p* < 0.001). In the pooled analysis of all ratings, the agreement between SCS and mSCS was K = 0.93 (*p* < 0.001). Overall, this shows an almost perfect agreement.

#### 3.5.3. Internal Consistency

The internal consistency of the ratings in the mSCS compared to the SCS (dichotomized) was tested using Cronbach’s alpha. An excellent internal consistency was found with Cronbach’s alpha values of 0.992 for the ratings of the MTB recommendations, 0.934 for the ratings of the LLM recommendations, and 0.964 for the pooled analysis of the ratings of the MTB and LLM recommendations.

After excluding item four, the only non-modified item, Cronbach’s alpha values of 0.989 were obtained for the ratings of the MTB recommendations, 0.926 for the ratings of the LLM recommendations, and 0.957 for the pooled analysis of the ratings of the MTB and LLM recommendations.

#### 3.5.4. Evaluation of Clinical Applicability of the mSCS Compared to the SCS

The mean Likert score for the clinical applicability of the mSCS items was 4.4 (SD = 0.70), while the score for the SCS items was 3 based on the upfront determination. There was a statistically significant increase in the Likert scores after the modification of the SCS (Z = −2.739, *p* = 0.006, n = 10), suggesting that the modification had a positive effect.

## 4. Discussion

The current study is intended as a preparatory investigation for the prospective, bicentric CONCORDIA Study. Hence, the specific LLMs to be used, the optimal input prompts for the LLMs, a sufficient measurement tool adapted to the specific research question, and the sample size calculation for the main study were developed based on 40 case scenarios that were discussed in a real MTB and subsequently compared with treatment recommendations from an LLM (ChatGPT-4).

MTBs consist of regular meetings of representatives of various clinical specialties, who discuss patient management and provide evidence-based and individual therapy decisions [30]. One can easily imagine that interdisciplinary exchange and various perspectives on patient cases ultimately lead to a more profound and higher-quality therapy decision. This effect seems to be particularly pronounced in tumor entities where established treatment options involve multiple specialties, such as surgery, medical oncology, radiation therapy, or nuclear medicine (as is often the case with GUCs). On the other hand, MTBs consume substantial personnel and financial resources to facilitate interdisciplinary exchange, which poses a genuine challenge in a world where both resources represent a true scarcity [8,9,10,31,32]. To investigate the effect of MTBs on patient outcomes, Huang et al. conducted a Meta-Analysis including 134,287 patients with various cancer entities from 59 studies. The authors found a significantly prolonged survival time (median survival time 30.2 months vs. 19 months) in patients managed by an MTB, suggesting that their implementation is likely worthwhile whenever possible [30].

LLMs are poised to take the scientific and clinical medical world by storm with their abilities in natural language processing, data analysis, predictive modeling, and generating evidence-based recommendations [1,2,3,4,5]. A particularly advantageous feature of LLMs is the ability to provide logical, coherent, and scientifically correct answers to various text questions, which is facilitated by deep learning algorithms and access to large-scale and up-to-date databases. It is precisely from this feature of LLMs that the research question of the CONCORDIA study is derived, namely whether LLMs can replace the complex, resource-intensive decision-making process of an MTB and ultimately generate a recommendation that is not inferior to that of the MTB.

Currently, there is limited evidence on the use of LLMs as auxiliary tools in MTBs for other cancer entities, and, to the best of our knowledge, no studies have been conducted in the context of GUC or compared the blinded recommendations of LLMs with those of an actual MTB [11,13,14,21]. One study investigated ChatGPT 3.5 and ChatGPT-4 as decision-making tools for 30 primary head and neck cancer cases [14]. Although the LLMs performed exceptionally well in providing clinical recommendations, explanations, and summaries, they suggested significantly more treatment options than the MTB and occasionally recommended incorrect guidelines. The authors concluded that while ChatGPT may support the MTB process, it is not capable of replacing it [14]. Another study by Stalp et al. evaluated ChatGPT 3.5s performance in suggesting treatments in 30 breast cancer cases [13]. While the therapy recommendations were judged to be mostly accurate, the quality of the recommendations was higher in primary cases, and complex patient histories posed a particular challenge for the LLM [13]. The study also demonstrated that the quality of recommendations is directly influenced by the prompt [13]. These findings align with another study by Griewing et al., which showed that using an extended input model further improved the quality of the LLMs’ recommendations [17]. In the current study, this issue was addressed by refining and optimizing the initial prompts for consistency and accuracy in a multi-stage process by the working group (ER, MH, DvW, and AK).

To assess the quality of AI-generated explanations, especially in the context of scientific model development, Holzinger et al. introduced the System Causability Scale (SCS) in 2020 [22]. The SCS quantifies explainability based on responses to 10 questions, each rated on a 5-point Likert scale. Its simplicity and status as a standardized tool make it highly useful for evaluating AI- and LLM-generated explanations [22]. A major limitation of this method, however, is that while the general nature of the questions allows the scale to be applied across various medical specialties, the individual items are not ideally suited to specifically assess the quality of a therapeutic recommendation. Our goal, therefore, was to modify the individual items so that they are precisely tailored to evaluating therapeutic recommendations for GUC patients within the scope of an MTB, enabling reviewers to provide an assessment that is both intuitive and accurate, as well as reproducible. Our results confirm strong validity, reliability (all aggregated Cohen’s K > 0.74), and internal consistency (all Cronbach’s Alpha > 0.9) for both scales. However, compared to the SCS, the mSCS demonstrated superior reliability, internal consistency, and clinical applicability (*p* < 0.01), leading us to conclude that this tool is highly suitable for assessing therapeutic recommendations within the framework of the planned CONCORDIA study.

Another critical step in the design of the CONCORDIA study was the determination of the optimal sample size. Based on the results of the current study, we took several factors into account: (1.) Power and effect size: In accordance with available recommendations on power analysis for clinical research studies, a statistician experienced in prospective study design (FZ) conducted the sample calculation based on a desired power set at 90% (beta = 0.1) and Bonferroni corrected alpha at 0.0125 (since a one-sided 2.5% level of significance is assumed and two LLMs will be compared against the real MTB). The expected variance was estimated based on the mean mSCS results (±STD) for the LLM and MTB, and the non-inferiority margin was set to 0.15, based on a four-round Delphi process, as described above. This led to a minimal required sample size of 87 patients. (2.) Adjustment for dropouts or missing information: To compensate for potential dropouts or missing data, the targeted patient number was increased by 25%, corresponding to 109 patients. To achieve an equal case distribution between the two study centers, the final patient number was set at 110. (3.) Sample representativeness: The current study reflects the real-world care in an actual MTB and encompasses the full spectrum of GUCs in their respective frequencies (16 prostate cancer, 9 urothelial cancer, 7 renal cell cancer, 4 testicular cancer, and 4 penile cancer cases). In the planned CONCORDIA study, case scenarios across different GUC entities will be distributed as accurately as possible by analyzing the actual frequency distribution of the real MTB cases for the two study centers.

Our findings underscore the potential of LLMs to aid clinical decision-making in oncology, particularly in resource-limited settings where access to multidisciplinary expertise may be constrained. The mSCS validated in this study could serve as a framework for integrating AI-supported recommendations in real-time clinical practice.

### Limitations

A limitation of both the current study and the upcoming CONCORDIA study is that, due to data privacy regulations from the local ethics committee, we are unable to discuss and compare real patient cases. To address this, we will create realistic case scenarios that are not based on actual patients. As previously mentioned, our goal is to align the distribution of these scenarios with the actual frequency distribution of the two MTBs across different GUC entities, ensuring a representative cohort for the CONCORDIA study. Additionally, when designing the cases, we are carefully preserving the structure of the original MTB cases to facilitate comparability between the two centers and ensure greater consistency in the case vignettes.

Despite its successful validation, the mSCS may encounter challenges in cases where LLM recommendations lack specificity or clinical nuance, especially in complex or ambiguous clinical scenarios. Further studies are warranted to refine these limitations in broader oncological contexts.

## 5. Conclusions

This study demonstrates the successful modification and validation of the SCS for evaluating AI-based therapeutic recommendations, specifically for patients with urological cancer. The validated mSCS offers clinicians a structured tool for assessing LLM recommendations in oncological practice, with potential applications in real-time decision-making and enhancing the reliability of AI-derived guidance.

Results from this preliminary work provide a robust methodological basis for the forthcoming non-inferiority trial CONCORDIA, which will compare treatment recommendations derived from LLMs and MTBs. The study’s design ensures a comprehensive assessment of AI’s role in clinical decision-making, with significant implications for future integration of AI in clinical oncology. The validated mSCS offers a valuable tool for evaluating LLM recommendations in this and similar trials. Ultimately, this work paves the way for advancing AI-supported cancer care.

## Figures and Tables

**Table 1:** Representation of the standardized sequence of input commands (prompts) into the large language model in German, along with the translation of the prompts into English.

Prompt-Original Input in German	Prompt-English Translation
Formuliere eine stichpunktartige Therapieempfehlung für den folgenden Patientenfall. Die Vortherapien und andere relevante Befunde sind im Fall enthalten. Beschränke dich hierbei nicht nur auf Medikamente, sondern beschreibe alle möglichen Therapieoptionen. Bitte benenne Therapien und Medikamente, falls du eine medikamentöse Therapie vorschlägst, konkret. Versuche, deine Empfehlung anhand der in Deutschland zugelassenen und leitliniengerechten Therapien zu treffen. Bitte benenne zudem explizit die aus deiner Sicht beste Therapieoption für den individuellen Patienten. Begrenze mit Deinen Antworten auf maximal 80 Wörter und orientiere Dich in ihnen an folgender Struktur: (1.) Präferierte Therapie-empfehlung (falls vorhanden), (2.) Therapie-alternativen, (3.) Begründung der Empfeh-lungen, (4.) Supportivmaßnahmen/ergänzende Therapien, (5.) Weiterführende Informationen/Erklärungen. Patientenfall:	Formulate a key point-based treatment recommendation for the following patient case. The previous therapies and other relevant findings are included in the case. Do not limit yourself to medication, but describe all possible treatment options. Please name therapies and medications specifically if you are suggesting drug therapy. Try to make your recommendation based on the therapies approved in Germany and in line with the guidelines. Please also explicitly state what you consider to be the best treatment option for the individual patient. Limit your answers to a maximum of 80 words and base them on the following structure: (1) Preferred therapy recommendation (if available), (2) Therapy alternatives, (3) Justification of the recommendations, (4) Supportive measures/supplementary therapies, (5) Further information/explanations. Patient case:

The prompt components are assigned to objectives: task (yellow), information (green), completeness and preferred option (gray), geographical categorization (blue), and formal requirements (purple).

**Table 3:** (**a**). Interrater reliability (K): SCS/mSCS Ratings concerning MTB vs. LLM recommendations. (**b**). Interrater reliability (ICC): SCS/mSCS Ratings concerning MTB vs. LLM recommendations.

(a)				
	Interrater Reliability SCS		Interrater Reliability mSCS	
	MTB	LLM	MTB	LLM
	K (*p*-Value)	K (*p*-Value)	K (*p*-Value)	K (*p*-Value)
Item 1	0.80 (<0.001)	0.70 (<0.001)	0.90 (<0.001)	0.70 (<0.001)
Item 2	0.95 (<0.001)	0.75 (<0.001)	1.00 (<0.001)	0.85 (<0.001)
Item 3	0.70 (<0.001)	0.75 (<0.001)	0.80 (<0.001)	0.65 (<0.001)
Item 4	1.00 (<0.001)	0.90 (<0.001)	1.00 (<0.001)	0.95 (<0.001)
Item 5	0.90 (<0.001)	0.70 (<0.001)	0.75 (<0.001)	0.70 (<0.001)
Item 6	0.95 (<0.001)	0.65 (<0.001)	1.00 (<0.001)	0.80 (<0.001)
Item 7	0.90 (<0.001)	0.70 (<0.001)	1.00 (<0.001)	0.80 (<0.001)
Item 8	0.85 (<0.001)	0.80 (<0.001)	1.00 (<0.001)	0.85 (<0.001)
Item 9	0.90 (<0.001)	0.85 (<0.001)	1.00 (<0.001)	0.95 (<0.001)
Item 10	1.00 (<0.001)	0.75 (<0.001)	1.00 (<0.001)	0.80 (<0.001)
**All Items**	**0.90 (<0.001)**	**0.74 (<0.001)**	**0.95 (<0.001)**	**0.81 (<** **0.** **001)**
(b)				
	Interrater Reliability SCS		Interrater Reliability mSCS	
	MTB	LLM	MTB	LLM
	ICC (*p*-Value)	ICC (*p*-Value)	ICC (*p*-Value)	ICC (*p*-Value)
Item 1	0.89 (<0.001)	0.83 (<0.001)	0.95 (<0.001)	0.83 (<0.001)
Item 2	0.98 (<0.001)	0.86 (<0.001)	1.00 (<0.001)	0.92 (<0.001)
Item 3	0.83 (<0.001)	0.86 (<0.001)	0.89 (<0.001)	0.79 (<0.001)
Item 4	1.00 (<0.001)	0.95 (<0.001)	1.00 (<0.001)	0.98 (<0.001)
Item 5	0.95 (<0.001)	0.83 (<0.001)	0.86 (<0.001)	0.83 (<0.001)
Item 6	0.98 (<0.001)	0.79 (<0.001)	1.00 (<0.001)	0.89 (<0.001)
Item 7	0.95 (<0.001)	0.83 (<0.001)	1.00 (<0.001)	0.89 (<0.001)
Item 8	0.92 (<0.001)	0.89 (<0.001)	1.00 (<0.001)	0.92 (<0.001)
Item 9	0.95 (<0.001)	0.92 (<0.001)	1.00 (<0.001)	0.98 (<0.001)
Item 10	1.00 (<0.001)	0.86 (<0.001)	1.00 (<0.001)	0.89 (<0.001)
**All Items**	**0.95 (<0.001)**	**0.85 (<0.001)**	**0.97 (<0.001)**	**0.89 (<** **0** **.001)**

## Data Availability

The raw data supporting the conclusions of this article will be made available by the authors upon request.
